# Promising synergistic interactions and mixture optimization of safranal, crocin, and crocetin from Moroccan *Crocus sativus* L. with enhanced antimicrobial activity

**DOI:** 10.3389/fphar.2026.1734075

**Published:** 2026-02-13

**Authors:** Abdellah Baraich, Ibrahim Sadougui, Amine Elbouzidi, Mounir Haddou, Mohamed Taibi, Abdessamad Beraich, Fatima Zahrae El Moussaoui, Omer M. Almarfadi, Ramzi A. Mothana, Mohammed F. Hawwal, Abdeslam Asehraou, Mohamed Addi, François Mesnard, Bassem Jaouadi, Ennouamane Saalaoui

**Affiliations:** 1 Laboratoire de Bioressources, Biotechnologie, Ethnopharmacologie et Santé (LBBES), Faculté des Sciences d’Oujda (FSO), Université Mohammed Premier (UMP), Oujda, Morocco; 2 Laboratoire d’Amélioration des Productions Agricoles, Biotechnologie et Environnement (LAPABE), Faculté des Sciences (FS), Université Mohammed Premier (UMP), Oujda, Morocco; 3 Laboratoire Environnement et Chimie Appliquée (LCAE), Equipe: Chimie Physique des Ressources Naturelles et des Procédés, Département de Chimie, Faculté des Sciences d’Oujda (FSO), Université Mohammed Premier (UMP), Oujda, Morocco; 4 Department of Pharmacognosy, College of Pharmacy, King Saud University, Riyadh, Saudi Arabia; 5 BIOPI-BioEcoAgro UMRT 1158 INRAE Université de Picardie Jules Verne, Amiens, France; 6 Laboratoire des Biotechnologies Microbiennes et Enzymatiques et Biomolécules (LBMEB), Centre de Biotechnologie de Sfax (CBS), Université de Sfax (USF), Sfax, Tunisia

**Keywords:** antimicrobial activity, crocetin, crocin, *Crocus sativus* L., minimum inhibitory concentration, mixturedesign, safranal, synergistic interaction

## Abstract

This study explores the antimicrobial activity of three principal bioactive constituents of *Crocus sativus* L.—safranal, crocin, and crocetin—using a simplex-centroid mixture design to evaluate their individual and synergistic effects. Twelve formulations were tested against *Staphylococcus aureus*, *Escherichia coli*, *Candida albicans*, and *Geotrichum candidum*. Minimum inhibitory concentrations (MICs) were determined via microdilution assays, and the results were fitted using special cubic regression models. The models exhibited strong predictive accuracy (R^2^ = 0.97–0.99). Safranal showed the highest individual activity against *S. aureus* (σ_1_ = 169.77, *p* = 0.0053), while crocin exerted the strongest effect on *E. coli* (σ_2_ = 166.63, *p* < 0.0001). Significant synergistic interactions were observed between crocin and crocetin for both bacterial strains (σ_23_ = −290.34 for *S. aureus*; −170.28 for *E. coli*). Ternary mixtures displayed superior efficacy compared to single compounds, producing the lowest MIC values across all pathogens. The optimal antibacterial formulation—33.31% safranal, 33.29% crocin, and 33.39% crocetin—yielded MICs of 41.14% *(v/v)* (predicted) and 39.5% ± 0.75% *(v/v)* (experimental) against *E. coli*. Against *S. aureus*, a blend of 27% safranal, 33% crocin, and 38% crocetin resulted in a predicted MIC of 42.15% *(v/v)*, confirmed experimentally at 38.13% ± 1.33% *(v/v)*. For *G. candidum*, the optimized mixture reached MIC values of 9.13% *(v/v)* (predicted) and 10.24% ± 2.05% *(v/v)* (observed). These findings demonstrate that synergistic combinations of saffron-derived metabolites markedly enhance antimicrobial potency. Moreover, mixture design modeling emerges as an effective predictive and optimization strategy for developing reproducible, plant-based antimicrobial formulations targeting antibiotic resistance.

## 1 Introduction

In recent years, traditional herbal medicine has garnered increasing interest among consumers, particularly when synthetic drug treatments prove ineffective or produce undesirable side effects. Phytotherapy remains one of the most widely practiced forms of alternative medicine, relying on natural products that are generally regarded as safe (Salmerón-Manzano et al., 2020). Typically, the pharmacological activity of plant extracts does not stem from a single active compound but rather results from the synergistic interaction among multiple bioactive constituents (Sofowora et al., 2013).

To better understand and optimize such synergistic interactions, mixture design methods have become increasingly important in natural product research. Unlike classical experimental approaches that examine one factor at a time, mixture design allows the systematic evaluation of different proportions of components within a mixture, enabling the identification of optimal combinations and synergistic effects. This statistical strategy is particularly suited for studying plant-derived compounds, whose biological activities often depend on complex interactions rather than the action of isolated molecules.

*Crocus sativus* (*C. sativus* L.), is a medicinal plant of remarkable significant therapeutic and nutritional significance. Because of its many uses as a spice, natural dye, and medicinal ingredient, it is a highly valued commercial crop that is widely grown throughout Europe, the Mediterranean region, and Central Asia. Saffron is a member of the Iridaceae family, which includes about 100 corm-propagated seasonal and permanent species. This species is triploid and infertile. Often referred to as “red gold,” saffron is the world’s most expensive spice, prized for its extensive applications in culinary, cosmetic, and medicinal industries (Sofowora et al., 2013).

The characteristic color and aroma of saffron are attributed to its bioactive compounds, notably crocin, crocetin, and safranal. These molecules have been the focus of numerous studies because of their potential health benefits, including antimicrobial, antioxidant, and anticancer properties. Despite numerous reports on the pharmacological actions of saffron compounds, comprehensive evaluations of their interactive effects remain scarce. Exploring such synergistic behaviors can provide valuable insights into how natural mixtures outperform isolated compounds. Owing to this broad spectrum of bioactivities, *C. sativus* is considered a promising candidate for the development of plant-based drugs targeting a variety of diseases (Roshanravan and Ghaffari, 2022). Despite extensive research on saffron’s pharmacological properties, the interactions among its major constituents remain poorly understood. A mechanistic exploration of these synergistic effects could help bridge the gap between empirical evidence and molecular-level understanding, paving the way for standardized phytopharmaceutical formulations.

The antimicrobial activity efficacy of saffron is attributed to safranal and crocin, while crocetin has recently emerged as a key co-factor that enhances biological activity through molecular synergy. These molecules exhibit physicochemical characteristics that enhance their antimicrobial efficacy safranal’s volatility and crocin’s water solubility facilitate their penetration and interaction with microbial cells, leading to their inhibition or even eradication (Naim et al., 2022).

This study aims to evaluate the antimicrobial efficacy of saffron’s three primary bioactive constituents crocin, crocetin, and safranal using an innovative experimental approach based on mixture design models. The objectives are twofold: first, to investigate the individual antimicrobial activity of each compound against a range of microbial pathogens; and second, to identify potential synergistic interactions that enhance their combined effectiveness. By systematically comparing the effects of pure and mixed formulations, this research seeks to elucidate favorable molecular interactions that could inform the development of more potent natural antimicrobial agents. The findings may contribute to novel therapeutic strategies for both the prevention of oxidative damage and the treatment of microbial infections, thereby advancing the medicinal and pharmacological potential of saffron.

## 2 Results

### 2.1 Optimization of antimicrobial effects through experimental mixture design

#### 2.1.1 Formulation design for antibacterial activity

The mixture design includes various combinations of the three studied molecules safranal, crocin, and crocetin along with the corresponding results obtained for each assay against *S. aureus* and *E. coli*. Twelve formulations were tested in a randomized order to minimize experimental bias. Each measurement was performed in triplicate, ensuring the reliability and reproducibility of the obtained data (Table 1).

**TABLE 1:** Different combinations selected according to the mixture design and the corresponding responses (MIC values of *S. aureus* and *E. coli*) observed for each experiment.

No.^a^	Safranal (M1)	Crocin (M2)	Crocetin (M3)	MIC (% *(v/v)*)^b^	
				*S. aureus*	*E. coli*
1	1	0	0	166.6	83.3
2	0	1	0	145.80	166.6
3	0	0	1	124.98	83.3
4	0.5	0.5	0	104.15	166.6
5	0.5	0	0.5	83.3	83.3
6	0	0.5	0.5	62.47	83.3
7	0.33	0.33	0.33	41.15	41.15
8	0.33	0.33	0.33	41.15	41.15
9	0.33	0.33	0.33	41.15	41.15
10	0.67	0.17	0.17	104.15	83.15
11	0.17	0.67	0.17	83.3	104.15
12	0.17	0.17	0.67	62.47	41.15

^a^ Experiments were performed after randomization.

^b^ The tests were conducted in three independent replicates and established as means ± SD.

#### 2.1.2 Statistical validation of the antibacterial model

The experimental data corresponding to each response variable were statistically analyzed to evaluate the adequacy of the selected special cubic model, which illustrates the correlation between the responses and the input factors.

##### 2.1.2.1 Staphylococcus aureus

**FIGURE 1:**
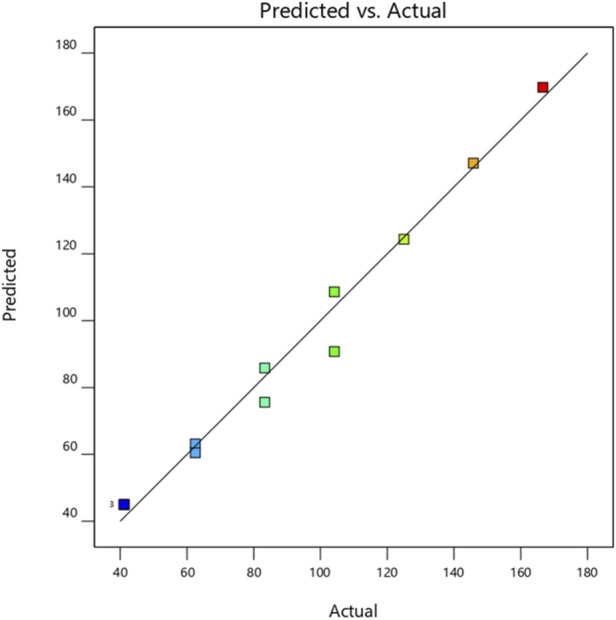
Plot of observed MIC values as a function of predicted MIC values for the *S. aureus* strain.

According to the analysis of variance presented in Table 2, the main effect of the regression is statistically significant for *S. aureus*, as indicated by a p-value less than 0.05 (*p* = 0.0003). The Fisher’s F-ratio is 48.78, underscoring the robustness and relevance of the proposed model. The goodness-of-fit was assessed using the coefficient of determination (R^2^), which reached a value of 0.98 for *S. aureus*, indicating an excellent explanatory power of the model. These results demonstrate a high level of agreement between the observed values and those predicted by the fitted model. This adequacy was further confirmed graphically in Figure 1, which displays a linear distribution of experimental values relative to the theoretical predictions.

**TABLE 2:** Analysis of variance for the proposed model applied to the mixture tested against *S. aureus*.

Source	Df	Su of square (SC)	Mean square (CM)	F-value	*p*-value
Regression (model)	6	19,013.89	3,168.98	48.78	<0.0003*
Residual (r)	5	324.82	64.96	​	​
Total	11	19,338.71	​	​	​
Coefficient of determination (R^2^)			**0.98**		​

Bold was used for values that are significant at *p* value < 0.05.

##### 2.1.2.2 Escherichia coli

**FIGURE 2:**
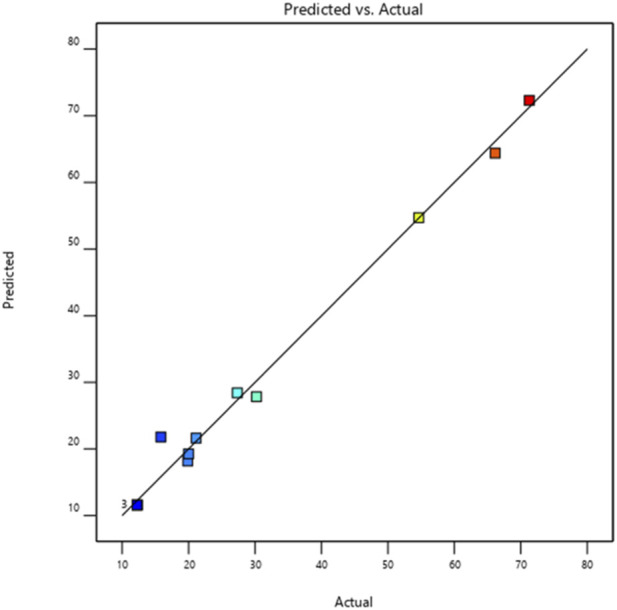
Plot of observed MIC values as a function of predicted MIC values for the *E. coli* strain.

According to the analysis of variance summarized in Table 3, the main effect of the regression model is highly significant for *E. coli*, as evidenced by a p-value well below the conventional significance threshold (*p* = 0.0001). The Fisher’s F-ratio (F = 222.85) further confirms the statistical robustness of the model. For *E. coli*, the validity of the model was supported by a high coefficient of determination (R^2^ = 0.99), indicating excellent predictive capability. These results reveal a strong correlation between the observed experimental data and the values estimated by the fitted model. This agreement was also visually confirmed in Figure 2, which displays a pronounced linear relationship between the measured and predicted values.

**TABLE 3:** Analysis of variance for the proposed model applied to the mixture tested against *E. coli.*

Source	Df	Sum of square (SS)	Mean square (MS)	F-value	*p*-value
Regression (model)	6	21,269.53	3,544.92	222.85	<0.0001*
Residual (r)	5	79.54	15.91	​	​
Total	11	21,349.07	​	​	​
Coefficient of determination (R^2^)			**0.99**		​

Bold was used for values that are significant at *p* value < 0.05.

### 2.2 Formulation design for antifungal activity

The mixture design incorporates various combinations of the three investigated compounds safranal, crocin, and crocetin along with the corresponding results obtained for each formulation tested against *C. albicans* and *G. candidum*. The twelve experimental runs were randomized to minimize potential experimental bias. Each formulation was tested in three independent replicates, ensuring the reliability and robustness of the data collected (Table 4).

**TABLE 4:** Different combinations selected according to the mixture design and the corresponding responses (MIC values of *C. albicans* and *G. candidum*) observed for each experiment.

No^a^	Safranal (M_1_)	Crocin (M_2_)	Crocetin (M_3_)	MIC (% *(v/v)*)^b^	
				*C. albicans*	*G. candidum*
1	1	0	0	166.6	104.12
2	0	1	0	145.8	166.6
3	0	0	1	124.98	83.3
4	0.5	0.5	0	104.15	83.3
5	0.5	0	0.5	83.3	83.3
6	0	0.5	0.5	62.47	166.6
7	0.333	0.333	0.333	41.15	20.8
8	0.333	0.333	0.333	41.15	20.8
9	0.333	0.333	0.333	41.15	20.8
10	0.667	0.167	0.167	104.15	41.65
11	0.167	0.667	0.167	83.3	83.3
12	0.167	0.167	0.667	62.47	62.47

^a^ Experiments were performed after randomization.

^b^ The tests were conducted in three independent replicates and established as means ± SD.

#### 2.2.1 Statistical validation of the antifungal model

The experimental data corresponding to the response of each strain were statistically analyzed to validate the selected special cubic model, which illustrates the correlation between the responses and the influencing factors.

##### 2.2.1.1 Candida albicans

**FIGURE 3:**
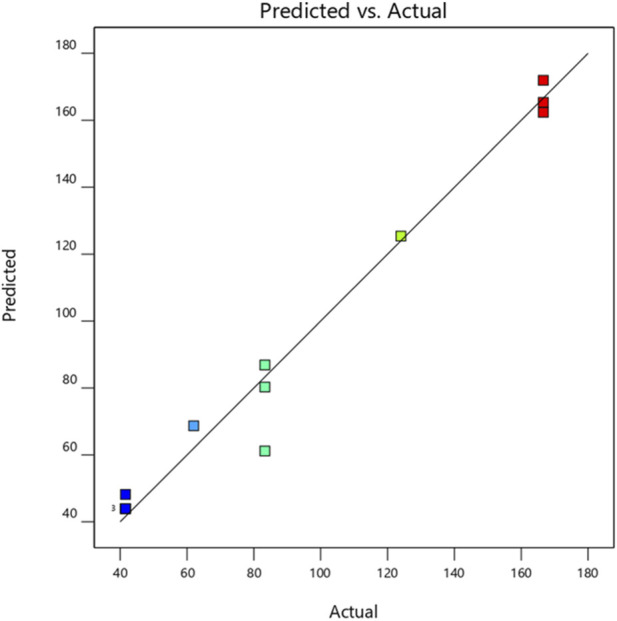
Plot of observed MIC values as a function of predicted MIC values for the *C. albicans* strain.

As shown by the analysis of variance in Table 5, the regression model exhibits a statistically significant main effect for *C. albicans* (*p* = 0.0006), confirming the robustness of the model (F = 35.48). The high coefficient of determination (R^2^ = 0.97) indicates an excellent correspondence between experimental and predicted values. This strong model fit was also visually supported by Figure 3, which displays a distinct linear relationship between measured and theoretical data.

**TABLE 5:** Analysis of variance for the regression model applied to the mixture evaluated against *Candida albicans*.

Source	Df	Sum of square (SS)	Mean square (MS)	F-value	*p*-value
Regression (model)	6	28,341.30	4,723.55	35.48	<0.0006*
Residual (r)	5	665.59	133.12	​	​
Total	11	29,006.89	​	​	​
Coefficient of determination (R^2^)			**0.97**		​

Bold was used for values that are significant at *p* value < 0.05.

##### 2.2.1.2 Geotrichum candidum

**FIGURE 4:**
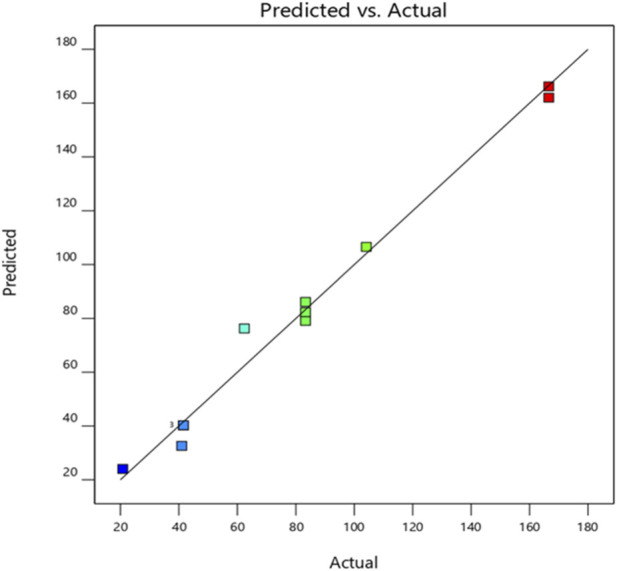
Plot of observed MIC values as a function of predicted MIC values for the *G. candidum* strain.

According to the analysis of variance presented in Table 6, the main effect of the regression model is statistically significant for *G. candidum*, as indicated by a *p*-value below the 0.05 threshold (*p* = 0.0002). The Fisher’s F-ratio (F = 62.71) confirms the relevance and statistical robustness of the model, while the coefficient of determination (R^2^ = 0.98) demonstrates an excellent fit to the experimental data. These findings highlight a strong correlation between the observed responses and the values predicted by the fitted model. This adequacy was further confirmed visually in Figure 4, which illustrates a clear linear relationship between the experimental data and the theoretical estimates.

**TABLE 6:** Analysis of variance (ANOVA) for the proposed model applied to the mixture tested against *G. candidum*.

Source	Df	Sum of square (SS)	Mean square (MS)	F-value	*p*-value
Regression (model)	6	24,993.42	4,165.57	62.71	<0.0002*
Residual (r)	5	332.11	66.42	​	​
Total	11	25,325.52	​	​	​
Coefficient of determination (R^2^)			**0.98**		​

Bold was used for values that are significant at *p* value < 0.05.

### 2.3 Components effects and adjusted models

#### 2.3.1 Antibacterial activity

The regression analysis presented in Table 7 provides valuable insight into the individual and interactive effects of safranal (A), crocin (B), and crocetin (C) on the minimum inhibitory concentration (MIC) against *S. aureus* and *E. coli*. All linear terms (σ_1_, σ_2_, σ_3_) were found to be statistically significant (*p* < 0.05) for both bacterial strains, indicating that each compound independently contributes to antibacterial activity. Among them, safranal exhibited the highest individual effect against *S. aureus* (σ_1_ = 169.77, *p* = 0.0053), while crocin had a slightly stronger influence on *E. coli* inhibition (σ_2_ = 166.63, *p* < 0.0001).

**TABLE 7:** Estimated regression coefficients and statistical significance for the effects of safranal, crocin, and crocetin on the MIC against *S. aureus* and *E. coli*.

Term	Coefficients	MIC (% *(v/v)*)			
		*S.aureus*		*E.coli*	
		Estimation	*p*-value	Estimation	*p*-value
Safranal (A)	σ_1_	169.77	0.0053*	85.22	0.0001 *
Crocin (B)	σ_2_	147.08	0.0053*	166.63	0.0001 *
Crocetin (C)	σ_3_	124.36	0.0053*	81.42	0.0001 *
Safranal* crocin (A*B)	σ_12_	−199.30	0.0038*	170.51	0.0003*
Safranal* crocetin (A*C)	σ_13_	−244.83	0.0015*	0.10	0.9959
Crocin* crocetin (B *C)	σ_23_	−290.34	0.0007*	−170.28	0.0003*
Safranal*Crocin* crocetin (A*B*C)	σ_123_	−552.85	0.0487 *	−1887.24	0.0001 *

-*Statistically signifcant at *p* < 0.05.

Bold was used for values that are significant at *p* value < 0.05.

The interaction effects offer deeper insight into potential synergistic or antagonistic relationships. Notably, the combination of crocin and crocetin (σ_23_) showed a strong negative coefficient for both *S. aureus* (−290.34, *p* = 0.0007) and *E. coli* (−170.28, *p* = 0.0003), suggesting significant synergistic antibacterial effects when these two compounds are used together. Similarly, the ternary interaction (σ_123_) was significant for both pathogens, especially for *E. coli* (−1887.24, *p* < 0.0001), indicating a highly enhanced inhibitory effect when all three constituents are combined.

In contrast, the interaction between safranal and crocetin (σ_13_) was only significant for *S. aureus* (−244.83, *p* = 0.0015) and showed no meaningful effect on *E. coli* (0.10, p = 0.9959), implying a species-specific interaction that does not contribute to *E. coli* inhibition.

Overall, these findings underscore the need to evaluate both single-compound efficacy and multi-component interactions to fully capture the antimicrobial potential of complex natural matrices. The observed synergistic effects may result from complementary physicochemical traits—safranal’s lipophilicity enhancing membrane disruption, crocin’s hydrophilicity improving intracellular diffusion, and crocetin mediating redox balance. The statistically significant negative interaction coefficients reflect synergistic behavior, where the combination of compounds yields a greater antibacterial effect than expected from their individual actions. Such findings support the potential of multi-compound strategies in antimicrobial formulations, particularly using natural products like *C. sativus* derivatives, which demonstrate selective and enhanced efficacy against Gram-positive and Gram-negative bacteria. The pronounced negative coefficients observed in binary and ternary interaction terms suggest a strong cooperative mechanism, possibly related to complementary physicochemical properties—such as differential solubility and membrane affinity—among the three molecules. This implies that structural diversity within a natural extract can be leveraged to achieve optimal antimicrobial performance.

#### 2.3.2 Antifungal activity

Table 8 presents the estimated regression coefficients and corresponding p-values for the antifungal effects of safranal, crocin, and crocetin individually and in combination on the minimum inhibitory concentrations (MICs) against *C. albicans* and *G. candidum*.

**TABLE 8:** Estimated regression coefficients and Statistical significance for the effects of safranal, crocin, and crocetin on the MIC against *C;albicans* and *G.candidum*.

Term	Coefficients	MIC (% *(v/v)*)			
		*C. albicans*		*G. candidum*	
		Estimation	*p*-value	Estimation	*p*-value
Safranal (A)	σ_1_	48.19	0.0005 *	104.19	0.0001 *
Crocin (B)	σ_2_	165.35	0.0005 *	164.78	0.0001 *
Crocetin (C)	σ_3_	80.29	0.0005 *	83.37	0.0001 *
Safranal* crocin (A*B)	σ_12_	260.69	0.0056 *	−211.71	0.0001*
Safranal* crocetin (A*C)	σ_13_	90.56	0.1674	−41.33	0.0757
Crocin* crocetin (B *C)	σ_23_	158.09	0.0372 *	163.12	0.0003 *
Safranal*Crocin* crocetin (A*B*C)	σ_123_	−2,986.27	0.0002 *	−2,384.71	0.0001 *

-*Statistically signifcant at *p* < 0.05.

Bold was used for values that are significant at *p* value < 0.05.

All three individual components exhibited statistically significant inhibitory effects (*p* < 0.001) against both fungal strains. Notably, crocin showed the highest positive coefficient values (165.35 for *C. albicans* and 164.78 for *G. candidum*), suggesting it is the most potent single agent in terms of antifungal activity among the tested compounds. Safranal and crocetin also contributed significantly, albeit to a lesser extent.

Regarding interaction effects, the binary interaction between safranal and crocin (σ_12_) had contrasting impacts: it significantly enhanced antifungal activity against *C. albicans* (positive coefficient = 260.69, *p* = 0.0056), but appeared to reduce efficacy against *G. candidum* (negative coefficient = −211.71, *p* < 0.0001), indicating a possible strain-specific antagonistic effect. The interaction between crocin and crocetin (σ_23_) was statistically significant for both fungi, suggesting a synergistic enhancement of antifungal activity when these two components are combined.

Interestingly, the three-way interaction term (σ_123_) exhibited a highly significant and strongly negative effect against both strains (−2,986.27 for *C. albicans* and −2,384.71 for *G. candidum*, *p* < 0.001), suggesting that the full ternary combination may result in a substantial antagonistic interaction, thereby diminishing overall antifungal efficacy. This highlights the importance of optimizing the ratios in multi-component formulations, as combining all three bioactives without precise balancing could counteract individual or binary synergies. Interestingly, this antagonism might be dose dependent. Future investigations could explore whether adjusting concentration ratios or applying sequential dosing could restore synergy, as seen in other plant-derived antifungal formulations.

Overall, these findings underscore the complex interplay between saffron-derived bioactives in modulating antifungal activity. While each compound demonstrates inherent antifungal potential, the combined effects particularly higher-order interactions must be carefully considered to avoid antagonism and to fully harness their therapeutic potential. The results support the rational design of plant-based antifungal formulations through optimized mixtures of specific constituents.

### 2.4 Optimization of formulation and desirability study

The optimization process, based on a mixture design approach, aimed to identify the most effective combination of variables, yielding superior outcomes compared to those obtained with the individual pure components. Although the statistically validated models are predictive, it is acknowledged that the optimal responses identified may not exactly replicate the results observed in the twelve experimental trials. Nonetheless, the models provide accurate estimations within the defined experimental space. Importantly, the combination of molecules consistently produced more favorable outcomes than any single component alone, highlighting the effectiveness of the mixture design strategy. To estimate the target values for optimal minimum inhibitory concentrations (MICs), the best experimental outcomes were used as reference benchmarks. The lowest MIC values recorded were 124.98% for *S. aureus*, 83.3% for *E. coli*, 145.8% for *C. albicans*, and 83.3% for *G. candidum*. Therefore, any formulation yielding MIC values equal to or lower than these thresholds was considered acceptable within the scope of this optimization framework.

#### 2.4.1 Mixture profile

This study highlights that the predicted optimal mixture exhibits stronger antimicrobial activity than any of the individual compounds across all tested microbial strains. The contour plots and three-dimensional surface graphs clearly illustrate the optimal combination of the three molecules, leading to a minimization of the MIC values. These visual tools effectively reveal the interactions between the independent variables, specifically the relative proportions of the components within the mixture. The dark blue regions on the plots correspond to the lowest MIC values, indicating high antimicrobial potency, while the gradient from green to red reflects decreasing effectiveness. Overall, the use of mixture design methodology proved to be a powerful approach for optimizing the proportions of the three compounds and identifying the formulation with the most pronounced antimicrobial effect.

##### 2.4.1.1 Efficacy of the molecular formulation against *S. aureus*

**FIGURE 5:**
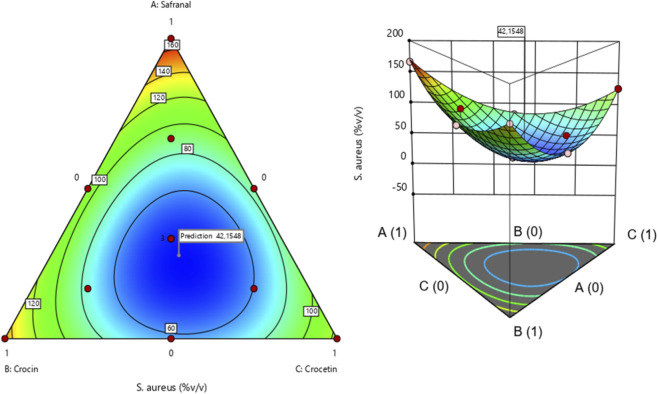
2D and 3D mixture diagrams showing variations in minimum inhibitory concentration (MIC) against *S. aureus* as a function of different proportions of safranal, crocin, and crocetin.

**FIGURE 6:**
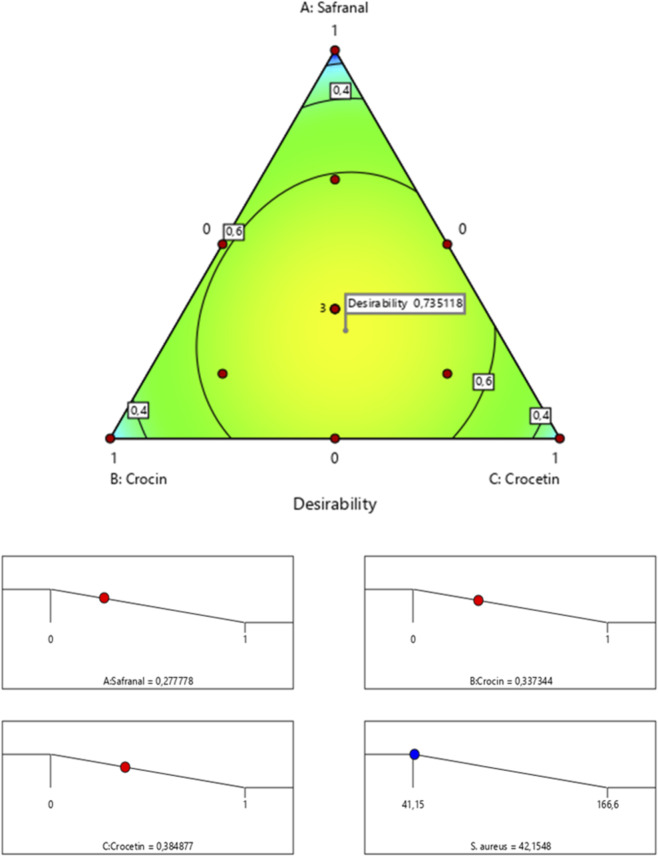
Desirability profile illustrating the optimal proportions of safranal, crocin, and crocetin for maximizing the antibacterial activity of the mixture against *S. aureus*.

The surface and contour response plots (Figure 5) illustrate the variation in minimum inhibitory concentration (MIC) against *Staphylococcus aureus* as a function of the relative proportions of the three tested molecules. Both the 2D and 3D graphical representations clearly demonstrate that the lowest MIC value approximately 42.15%, can only be achieved through a ternary combination of safranal, crocin, and crocetin. This finding is further supported by the desirability function analysis (Figure 6), which identifies an optimal formulation consisting of 27.77% safranal, 33.73% crocin, and 38.48% crocetin. This mixture is predicted to achieve the target MIC value of 42.15% with a corresponding desirability score of 73.77%, confirming the effectiveness of the blend in enhancing antimicrobial activity against *S. aureus*.

##### 2.4.1.2 Efficacy of the molecular formulation against *E. coli*

**FIGURE 7:**
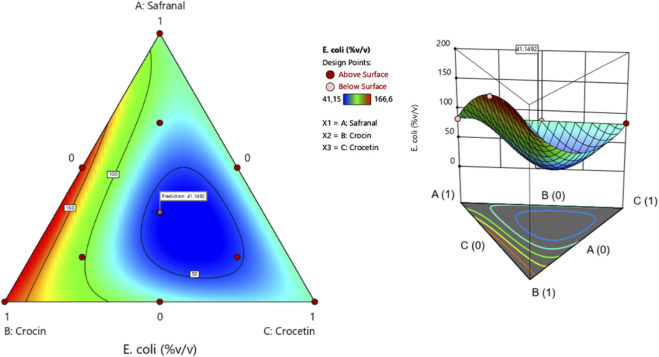
2D and 3D mixture diagrams showing variations in minimum inhibitory concentration (MIC) against *E. coli* as a function of different proportions of safranal, crocin, and crocetin.

**FIGURE 8:**
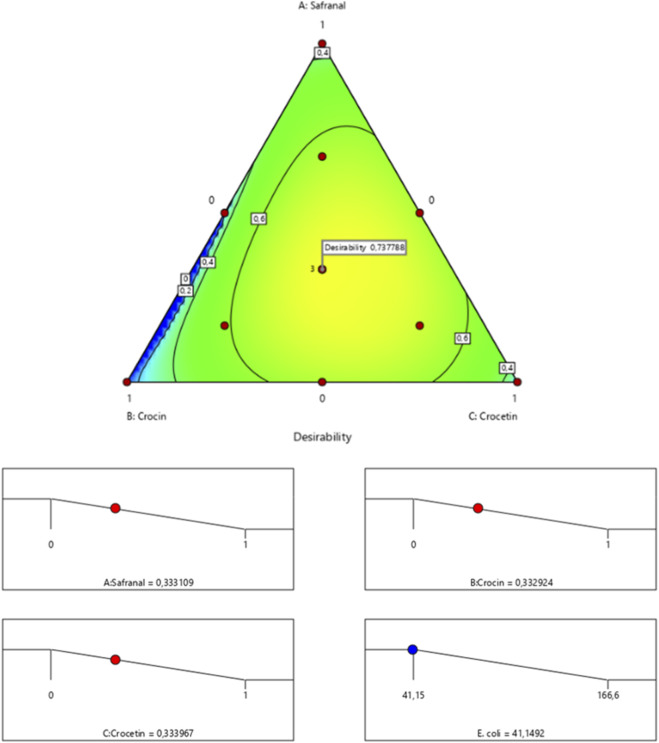
Desirability profile indicating the optimal proportions of safranal, crocin, and crocetin required to maximize the antibacterial activity of the mixture against *E. coli*.

The surface and contour response plots (Figure 7) illustrate the variation in minimum inhibitory concentration (MIC) against *Escherichia coli* as a function of the varying proportions of the three tested compounds. The 2D and 3D graphical representations reveal that the lowest MIC value, estimated at 41.14%, is only achieved through the simultaneous combination of safranal, crocin, and crocetin. This result is further supported by the desirability function analysis (Figure 8), which confirms that an optimal formulation comprising 33.31% safranal, 33.29% crocin, and 33.39% crocetin can achieve the target MIC value of 41.14% with an overall desirability score of 73.77%. These findings highlight the importance of balanced ternary mixtures in enhancing antibacterial efficacy against *E. coli*.

##### 2.4.1.3 Efficacy of the molecular formulation against *C. albicans*

**FIGURE 9:**
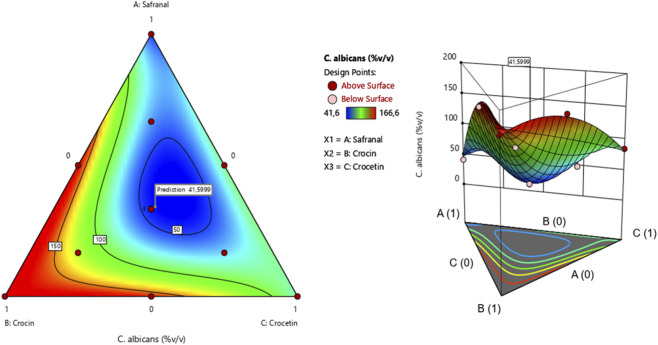
2D and 3D mixture diagrams illustrating variations in minimum inhibitory concentration (MIC) against *C. albicans* as a function of the relative proportions of safranal, crocin, and crocetin.

**FIGURE 10:**
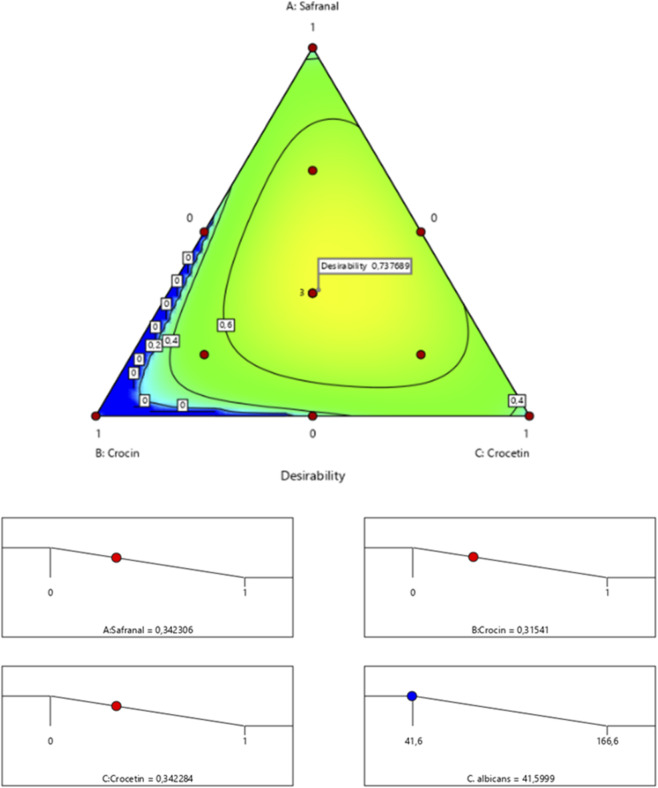
Desirability profile showing the optimal proportions of safranal, crocin, and crocetin for maximizing the antifungal activity of the mixture against *C. albicans*.

The surface and contour response plots (Figure 9) illustrate the variation in minimum inhibitory concentration (MIC) against *Candida* albicans as a function of the relative proportions of the three tested compounds. As shown in Table 4, the experimental MIC values ranged from 41.6% to 166.6%. The 2D and 3D graphical representations clearly indicate that the lowest MIC value estimated at 41.59% can only be achieved through a synergistic interaction among safranal, crocin, and crocetin. This finding is further supported by the desirability function analysis (Figure 10), which identifies an optimal mixture consisting of 34.23% safranal, 31.54% crocin, and 34.22% crocetin. This combination yields a predicted MIC of 41.59%, with a corresponding overall desirability score of 73.76%, highlighting the enhanced antifungal potential of the ternary formulation against *C. albicans*.

##### 2.4.1.4 Efficacy of the molecular formulation against *G. candidum*

**FIGURE 11:**
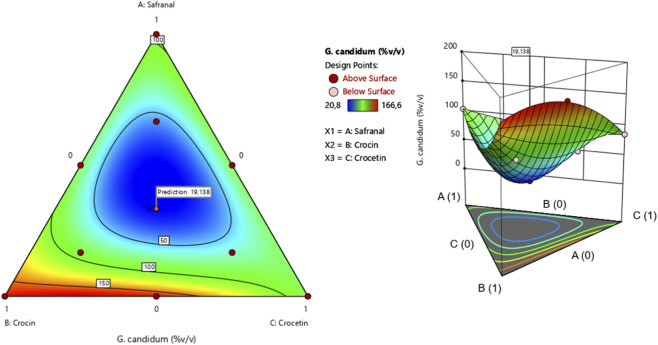
2D and 3D mixture diagrams illustrating variations in minimum inhibitory concentration (MIC) against *G. candidum* as a function of the relative proportions of safranal, crocin, and crocetin.

**FIGURE 12:**
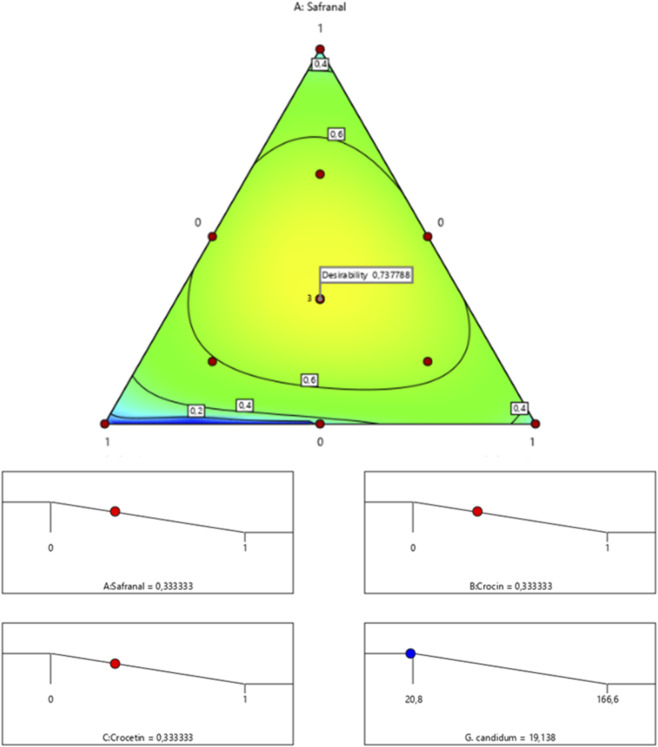
Desirability profile showing the optimal proportions of safranal, crocin, and crocetin for maximizing the antifungal activity of the mixture against *G. candidum*.

The surface and contour response plots (Figure 11) illustrate the variation in minimum inhibitory concentration (MIC) against *G. candidum* as a function of the relative proportions of the three tested compounds. The 2D and 3D graphical representations clearly show that the lowest MIC value estimated at 19.13% can only be achieved through a synergistic interaction among safranal, crocin, and crocetin. This observation is further supported by the desirability function analysis (Figure 12), which identifies an optimal mixture composed of 33.33% safranal, 33.33% crocin, and 33.33% crocetin. This balanced ternary formulation yields the predicted MIC of 19.13%, with an overall desirability score of 73.77%, highlighting the enhanced antifungal efficacy of the combination against *G. candidum*.

### 2.5 Simultaneous optimization of all responses

#### 2.5.1 Antibacterial activity

**FIGURE 13:**
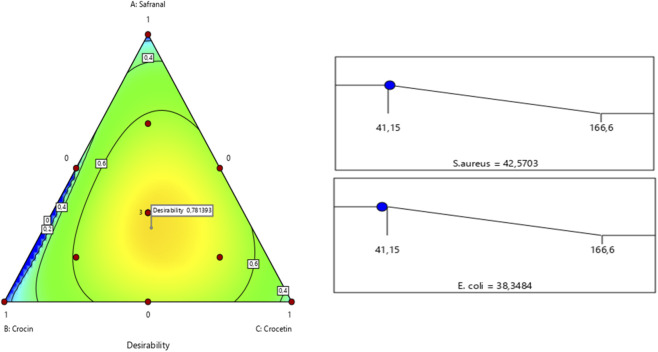
Mixture profile tested simultaneously against *S. aureus* and *E. coli*.

The outcomes of simultaneous optimization are clearly depicted through contour plots illustrating the antimicrobial responses against *S. aureus* and *E. coli*, as a function of the relative proportions of the investigated compounds. Notably, the optimized formulations exhibited superior antimicrobial activity compared to the individual components, thereby confirming the enhanced efficacy of the combined mixture. Achieving a satisfactory minimum inhibitory concentration (MIC) for both bacterial strains required a precise balance in the proportions of safranal, crocin, and crocetin. Specifically, a ternary mixture consisting of 27.66% safranal, 35% crocin, and 37.32% crocetin simultaneously achieved MIC values of 42.57% against *S. aureus* and 38.34% against *E. coli* (Figure 13).

#### 2.5.2 Antifungal activity

**FIGURE 14:**
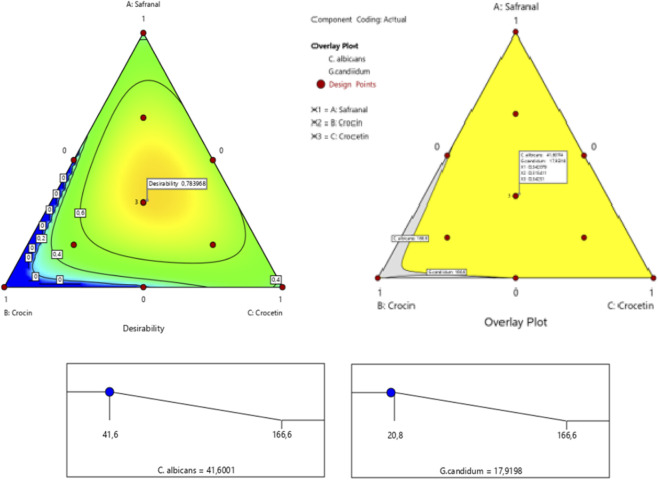
Mixture profile tested simultaneously against *C. albicans* and *G. candidum*.

Simultaneous optimization results are effectively visualized through contour plots depicting antimicrobial responses against *C. albicans* and *G. candidum*, based on varying proportions of the studied bioactive compounds. The optimized formulations demonstrated markedly enhanced antimicrobial efficacy relative to the individual components, highlighting the synergistic potential of the ternary mixture. Achieving satisfactory minimum inhibitory concentrations (MICs) for both fungal strains required a finely tuned balance between the three constituents: safranal, crocin, and crocetin. Notably, a mixture containing 27.66% safranal, 35% crocin, and 37.32% crocetin resulted in simultaneous MIC reductions to 42.57% against *C. albicans* and 38.34% against *G. candidum* (Figure 14).

### 2.6 Experimental verification of the assumed model

Table 8 presents both predicted and experimentally measured minimum inhibitory concentrations (MICs) for various microbial strains, based on optimized mixtures of three bioactive extracts: safranal, crocin, and crocetin. The specific proportions of each component were adjusted to maximize antimicrobial activity, and model predictions were compared against values obtained from triplicate experimental assays.

For *S. aureus*, the experimental MIC (38.13% ± 1.33%) was slightly lower than the predicted value (42.15% ± 0.000%), suggesting that the antimicrobial effect of the mixture may have been slightly underestimated by the model. This minor deviation could be attributed to experimental variability, natural fluctuations in strain susceptibility, or additional synergistic mechanisms not fully captured by the statistical model under the specific experimental conditions tested. Overall, it highlights the robustness of the mixture’s activity against this Gram-positive strain.

In the case of *E. coli*, experimental (39.5% ± 0.75%) and predicted (41.14% ± 0.000%) values were closely aligned. The mixture consisted of equal proportions of the three extracts (33% each), indicating that a balanced formulation can achieve effective inhibition of this Gram-negative bacterium. The small discrepancy supports the model’s validity and reinforces the potential utility of uniform extract compositions for bacterial inhibition.

Conversely, for *C. albicans*, a fungal pathogen, the experimental MIC (44.35% ± 2.54%) exceeded the predicted value (41.59% ± 0.000%). This difference may reflect intrinsic variability in fungal susceptibility or limitations of the model in capturing the biological complexity of eukaryotic organisms. Such findings underscore the importance of further refining predictive algorithms, particularly when targeting fungi with potentially more heterogeneous responses.

Regarding *G. candidum*, another fungal strain, the experimental MIC (10.24% ± 2.05%) was slightly higher than predicted (9.13% ± 0.000%). Nevertheless, the overall agreement remains satisfactory, particularly considering the balanced formulation (33% of each component). This result supports the model’s reliability in predicting antimicrobial outcomes even in eukaryotic systems, while also pointing to some experimental variability.

In summary, the data in Table 9 validate the overall reliability of the predictive model, while also revealing strain-specific variations that warrant further investigation. These findings emphasize the need to better characterize biological interactions among extract components and to refine modeling approaches to account for microbial diversity. They also offer promising perspectives for the development of natural compound-based antimicrobial formulations, with potential applications in pharmaceutical and food preservation contexts.

**TABLE 9:** Expected and observed responses for the test point that the best-fit mixes were able to achieve.

Strains	Predi/Exp	MIC (% *(v/v)*)	Proportions of each extract (%)		
			Safranal	Crocin	Crocetin
*S. aureus*	Predi^a^	42.15 ± 0.000	27%	33%	38%
	Exp^b^	38.13 ± 1.33			
*E. coli*	Predi^a^	41.14 ± 0.000	33%	33%	33%
	Exp^b^	39.5 ± 0.75			
*C. albicans*	Predi^a^	41.59 ± 0.000	34%	31%	34%
	Exp^b^	44.35 ± 2.54			
*G. candidum*	Predi^a^	9.13 ± 0.000	33%	33%	33%
	Exp^b^	10.24 ± 2.05			

^a^ The experimental value is represented as the average of three replicates.

^b^ The expected value includes the response’s standard deviation (±SD), as determined by the model.

## 3 Discussion

The integration of statistical modeling and antimicrobial screening provides a comprehensive framework for identifying optimal compound ratios, linking empirical observations with quantitative design principles. Traditional medicinal uses of saffron have been associated with its antimicrobial properties, primarily attributed to its bioactive constituents such as safranal, crocin, and crocetin. In the present study, the minimum inhibitory concentrations (MICs) of these isolated compounds were evaluated against two bacterial strains (*S. aureus* and *E. coli*) and two fungal strains.

The results reveal a significant variation in the antibacterial efficacy of the three compounds when tested individually and in combination against *S*. *aureus* and *E. coli*. Safranal exhibited an MIC of 83.3 *μ*g/mL against *E. coli* and 166.6 *μ*g/mL against *S. aureus*. These findings align with those reported by Miguel et al. (2011), who demonstrated the antimicrobial potential of safranal against several pathogens. They reported MIC values of approximately 8,000 *μ*g/mL for *E. coli*, and between 4,000 and 8,000 *μ*g/mL for *S. aureus*, thereby confirming its inhibitory capacity at relatively high concentrations (Miguel et al., 2011).

Crocin showed MIC values of 145.8 *μ*g/mL and 166.6 *μ*g/mL against *S. aureus* and *E. coli*, respectively. These results are consistent with Hussein et al. (2018), who reported strong antibacterial activity of crocin, with MICs of 100 *μ*g/mL against both bacterial strains (Hussein et al., 2018).

Crocetin displayed antibacterial activity with an MIC of 124.98 *μ*g/mL against *S. aureus* and 83.3 *μ*g/mL against *E. coli*. However, Paramanya et al. (2024) reported that crocetin inhibited *S. aureus* only at concentrations exceeding 800 *μ*g/mL, although it exerted significant anti-biofilm activity at concentrations as low as 2 *μ*g/mL.

Binary combinations of these compounds demonstrated enhanced antibacterial activity, as evidenced by reduced MIC values compared to the individual compounds. This synergistic effect was particularly pronounced against Gram-positive bacteria, which is consistent with structural differences in bacterial cell walls; Gram-negative bacteria possess an outer membrane that limits permeability, whereas Gram-positive bacteria have a thick peptidoglycan layer without an outer membrane (Chouhan et al., 2017).

Ternary combinations (experiments 7, 8, and 9) exhibited the highest antibacterial efficacy, with MIC values as low as 41.15 *μ*g/mL against both Gram-positive (*S. aureus*) and Gram-negative (*E. coli*) strains. These findings strongly support the presence of a synergistic interaction among the three saffron derived compounds (Samiee et al., 2017). Such synergy may arise from multi-target mechanisms, where different molecular structures simultaneously disrupt cell membrane integrity, interfere with metabolic enzymes, and inhibit oxidative stress pathways—yielding a combined antimicrobial impact greater than the sum of individual effects.

Our findings are in line with those reported by Jeddi et al. (2023), who used a simplex-centroid mixture design to optimize the antibacterial effects of three chemically characterized essential oils: *Eucalyptus polybractea*, *Ormenis mixta*, and *Lavandula burnatii*. Like our study, they demonstrated that ternary formulations resulted in significantly higher antimicrobial activity than individual oils or binary mixtures, indicating synergistic effects. For instance, their MIC values against *E. coli* and *S. aureus* were reduced to 0.5% and 0.125%, respectively, when essential oils were mixed in equal proportions substantially lower than the MICs of the individual oils (Jeddi et al., 2023).

Although the precise antibacterial mechanism of saffron’s bioactive compounds remains unclear, MaSon et al. (2017) showed that safranal exerts potent antibacterial effects by inhibiting ATP synthase, a key enzyme involved in bacterial energy production. By blocking ATP synthesis, safranal reduces the energy available for bacterial survival and proliferation, thus impairing cell growth (MaSon et al., 2017). The synergistic effects observed in this study likely arise from the complementary and multi-targeted actions of safranal, crocin, and crocetin, supported by several mechanistic hypotheses in the literature. Crocin and crocetin can modulate intracellular redox balance in eukaryotic systems. Crocetin activates the Nrf2/HO-1 antioxidant pathway, while crocin reduces ROS accumulation in LPS-stimulated microglial cells (Wen et al., 2021; Nam et al., 2010). Although similar redox effects have not yet been formally demonstrated in bacterial cells, their ability to influence oxidative homeostasis suggests that they may alter microbial stress responses or sensitize cells to other antimicrobial insults. These findings indicate that the synergy among safranal, crocin, and crocetin likely arises from the convergence of multiple mechanisms, including membrane destabilization, impaired energy metabolism, and redox modulation, rather than a single dominant target.

Despite these promising findings, several important limitations should be considered, particularly the lack of cytotoxicity or biocompatibility assays in the present study. Although *Crocus sativus* and its constituents are generally regarded as safe and have shown high LD_50_ values in rodent models, the specific toxicity profile of the concentrated ternary mixture remains unknown (Butnariu et al., 2022; Khazdair et al., 2015; Bahmani et al., 2014). Additionally, previous reports indicate that safranal may exert dose-dependent cytotoxic effects in mammalian cells, while crocin and crocetin exhibit variable safety profiles depending on the dose and cell type (Jabini et al., 2017; Zhao et al., 2021). This underscores the need for a comprehensive safety evaluation. Such an assessment should include MTT or LDH assays, hemolysis tests, and selectivity index calculations to establish the therapeutic window and ensure that antimicrobial efficacy is not accompanied by unacceptable toxicity to host cells.

## 4 Materials and methods

### 4.1 Tested molecules

The three bioactive compounds investigated in this study—crocin (PHL80391; Sigma-Aldrich, St. Louis, MO, USA), crocetin (SML3255; Sigma-Aldrich, St. Louis, MO, USA), and safranal (W338907; Sigma-Aldrich, St. Louis, MO, USA)—were purchased from Sigma-Aldrich, a supplier of certified analytical-grade reagents. The specified purity levels were ≥98% for crocin and crocetin and ≥90% for safranal.

### 4.2 Antimicrobial assays

#### 4.2.1 Microorganisms

To evaluate the antibacterial and antifungal activities of the compounds safranal, crocin, and crocetin, a panel of clinically and industrially relevant microorganisms was selected. The bacterial strains included *Staphylococcus aureus* ATCC 6538, a Gram-positive bacterium frequently associated with nosocomial infections and notable for its rising antibiotic resistance, along with *Escherichia coli* ATCC 10536, a Gram-negative organism of notable clinical importance, particularly in urinary tract and gastrointestinal infections (Baraich et al., 2025). In addition, two fungal species were included: *Candida albicans*, the most prevalent opportunistic fungal pathogen in humans, and *Geotrichum candidum*, a spoilage microorganism of economic relevance in the food industry (Eliskases-Lechner et al., 2025; Macias et al., 2022). All microbial strains were obtained from the Laboratory of bioresources, biotechnology, ethnopharmacology and health of the Faculty of Sciences, Oujda, Morocco.

Bacterial strains were reactivated by streaking with a loop onto Luria-Bertani (LB; BIOKAR, France) agar plates, followed by incubation at 37 °C for 20 h (Bertani, 1951). Fresh bacterial suspensions were then prepared in 10 mL of sterile physiological saline (NaCl; Sigma-Aldrich), with turbidity adjusted to a 0.5 McFarland standard, corresponding to approximately 10^6^ CFU/mL, in accordance with the guidelines of the National Committee for Clinical Laboratory Standards (NCCLS, USA) (Wayne, 2002; Haddou et al., 2024).

*G*. *candidum* was cultured on Potato Dextrose Agar (PDA; BIOKAR, France) at 25 °C for 7 days. Following incubation, the spore concentration was adjusted to 2 × 10^6^ spores/mL using a hemocytometer (Thoma chamber) (Kawtharani, 2021). Similarly, *C. albicans* was grown on Yeast Extract Peptone Dextrose (YPD; BIOKAR, France) medium at 25 °C for 48 h, and the cell density was standardized to 10^6^ cells/mL (Schaller et al., 2005).

#### 4.2.2 Determination of MIC

The minimum inhibitory concentrations (MICs) of the tested compounds were determined following a modified microdilution protocol based on the methodology described by Baraich et *al*. (Baraich et al., 2025). The samples underwent two-fold serial dilutions, yielding final concentrations ranging from 16% to 0.060% (*w/v*). These dilutions were prepared in Mueller-Hinton (M-H; BIOKAR, France) broth supplemented with 0.15% agar to enhance viscosity and minimize compound diffusion, and 50 *µ*L of each dilution was dispensed into sterile 96-well microtiter plates. Subsequently, 50 *µ*L of the bacterial suspension standardized to the appropriate inoculum density was added to each well, resulting in a final volume of 100 *µ*L per well. The Minimal Inhibitory Concentration (MIC) values were expressed as % (v/v), as all test samples were prepared by serial volumetric dilutions of liquid solutions of safranal, crocin, and crocetin. This unit is widely used in antimicrobial assays involving natural compounds and volatile molecules where volumetric proportions better represent the active concentration (Griffin et al., 1999; Karakaya et al., 2019). Wells containing only M-H broth with 0.15% agar and bacterial inoculum served as growth controls, while wells with medium alone functioned as sterility controls. After incubation at 37 °C for 24 h, 12 *µ*L of resazurin solution (0.017%, *w/v*) was added to each well as a redox indicator to assess bacterial viability. The MIC was defined as the lowest concentration of the test compound that inhibited visible bacterial growth, indicated by the retention of the blue color of resazurin. All experiments were performed in triplicate to ensure reproducibility and statistical reliability of the results.

#### 4.2.3 Normalization and interpretation of MIC values in % (v/v) units

For each tested compound (safranal, crocin, and crocetin), a working stock solution was prepared at a concentration of 6 mg/mL (equivalent to 100% for dilution purposes). MIC assays were then performed by serial two-fold volumetric dilutions of this stock solution in Mueller–Hinton broth to obtain final assay concentrations ranging from 0.06% to 16% (*v/v*). However, for the purpose of the mixture design analysis, and in order to normalize data across different molecular types and solubilities, the experimental MICs were recalculated and expressed as relative percentages of the stock solution required to inhibit microbial growth. Therefore, values exceeding 100% (*v/v*) represent the theoretical equivalent volume of stock solution required to reach full inhibition, when no complete inhibition was achieved within the tested range. These values thus indicate low antimicrobial potency of the pure compound rather than actual experimental pipetting beyond the 100% limit. In practice, all tested volumes were within the physical range of the assay and never exceeded the 100% working stock.

### 4.3 Experimental design

#### 4.3.1 Mixture design

The simplex-centroid mixture design was employed to systematically evaluate both linear and interaction effects among the three components. The design included vertex points representing pure components, edge midpoints corresponding to binary mixtures, and a central point representing the ternary mixture. To enhance model precision in critical formulation regions, additional supplementary points (67:16:17 ratios) were incorporated. This optimized experimental strategy enables efficient assessment of potential synergistic or antagonistic interactions between components, while minimizing the number of experimental runs aligning with established principles of mixture design methodology (Benkhaira et al., 2023). This approach not only ensures statistical robustness but also provides insights into the potential non-linear effects and synergistic behaviors that may arise when bioactive components interact in varying ratios. As detailed in Table 10, the proportions of each compound varied from 0 to 1, with the sum of all component fractions constrained to unity, in accordance with the foundational rules of mixture experiments (Elbouzidi et al., 2025). This systematic approach ensured efficient exploration of the formulation space, providing statistically reliable insights into potential synergistic or antagonistic interactions among the tested bioactives.

**TABLE 10:** Identification of independent variables used in the mixture.

Molecules	Coded variables	Level -	Level +
Safranal	M_1_	0	1
Crocin	M_2_	0	1
Crocetin	M_3_	0	1
Sum of proportions		**1**	

Bold was used for values that are significant at *p* value < 0.05.

#### 4.3.2 Experimental matrix and mathematical model

**FIGURE 15:**
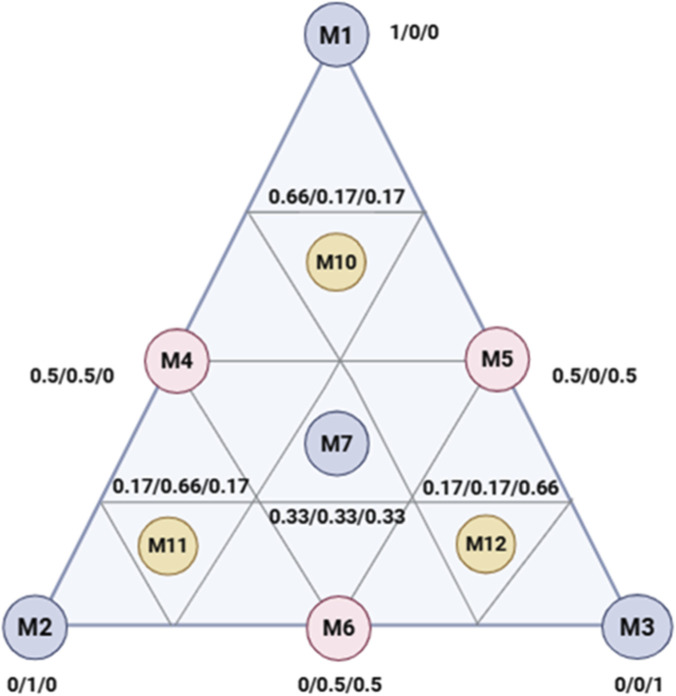
Equilateral triangle of the arrangement of mixtures using the simplex centroid design method. M1: Safranal; M2: Crocin; M3: M1-10: experimental combinations.

In this study, a total of 10 experimental formulations were designed and mapped within a simplex coordinate system represented by an equilateral triangle (Figure 15). The vertices of the triangle (M1, M2, and M3) correspond to the three pure components (1/0/0, 0/1/0, and 0/0/1, respectively). The midpoints of the edges (M4, M5, and M6) represent binary mixtures at equal proportions (0.5/0.5/0.0), while the centroid (M7) denotes the ternary mixture with equal proportions of each component (0.33/0.33/0.33). Each experimental run was conducted in triplicate to ensure statistical robustness. Additionally, three supplementary control points (M10, M11, and M12) were introduced to explore ternary mixtures with skewed proportions (0.67/0.16/0.16), allowing for finer resolution in regions of potential synergistic interaction. A cubic polynomial model was applied to describe the experimental responses as functions of the mixture components. The mathematical expression of the model is presented in Equation 1.

$$Y={\sigma_{1}M}_{1}+{\sigma_{2}M}_{2}+{\sigma_{3}M}_{3}+\sigma_{12}M_{1}X_{2}\;{+\sigma }_{13}M_{1}X_{3}+\sigma_{23}M_{2}M_{3}+\sigma_{123}M_{1}M_{2}M_{3}+ɛ$$ (1)

In this context, Y denotes the experimental response, specifically measured as the half-maximal inhibitory concentration (IC_50_, expressed in *µ*g/mL). The coefficients σ_1_, σ_2_, and σ_3_ represent the linear (main) effects associated with each of the three individual components. The interaction terms σ_12_, σ_13_, and σ_23_ capture the binary interactions between pairs of components, while σ_123_ accounts for the ternary interaction among all three constituents. The term ɛ corresponds to the residual error, representing the variability not explained by the model.

### 4.4 Statistical analysis

The experimental design, along with statistical and graphical analyses, was conducted using Design Expert software (version 12). Results are expressed as means ± standard deviation (SD), based on three independent replicates (n = 3), in accordance with established practices (Baraich et al., 2025). To assess the significance of the fitted models, analysis of variance (ANOVA) was applied. The F-test (F-ratio), defined as the ratio between the mean square of the regression and that of the residuals, was interpreted at a 95% confidence level. A high F-ratio indicates that the model accounts for a substantial portion of the variability observed in the experimental data, thereby confirming its predictive relevance. In addition, a comparison between the lack-of-fit and pure error mean squares was performed to evaluate the model’s adequacy in describing the data. A notably high ratio between these two parameters suggests that the model does not sufficiently capture the experimental variability, pointing to a potential need for refinement (Fadil and Farah, 2023). To further assess model performance, the coefficient of determination (R^2^) was calculated, reflecting the proportion of variance explained by the model. An R^2^ value close to 1 denotes a strong agreement between predicted and observed values. Moreover, Student’s t-test was employed to determine the statistical significance of individual regression coefficients, enabling validation of the contributions of each term within the model.

## 5 Conclusion

This work explored the antimicrobial potential of three key compounds naturally present in *C. sativus* L. safranal, crocin, and crocetin using a simplex-centroid mixture design. Rather than acting in isolation, these molecules showed enhanced antimicrobial effects when combined in specific proportions. The statistical models, supported by experimental data, clearly indicated that synergistic interactions play a central role in boosting their efficacy. Ternary mixtures were particularly effective, consistently producing the lowest MIC values against a panel of bacterial and fungal strains, including *S. aureus*, *E. coli*, *C. albicans*, and *G. candidum*.

The mixture design approach proved essential for identifying synergistic relationships among safranal, crocin, and crocetin. By systematically varying component proportions, this method captured both linear and nonlinear interactions, enabling prediction of optimal antimicrobial combinations. The strong agreement between experimental and predicted MICs confirms the model’s reliability. This approach, increasingly used in essential oil and phytochemical optimization, allows researchers to fine-tune formulations that maximize bioactivity while minimizing experimental workload. Hence, mixture design emerges as a powerful predictive tool for rationally developing synergistic, plant-based antimicrobial formulations. This strategy offers promising perspectives for the development of plant-derived antimicrobial agents, especially in the context of growing antibiotic resistance. The integration of phytochemical synergy analysis with computational mixture optimization represents a promising avenue for next-generation antimicrobial discovery. Future studies combining *in vitro* and *in silico* approaches could further refine predictive accuracy and accelerate the translation of natural compounds into clinically viable agents. Future work should integrate *in vitro* synergy testing with computational modeling and *in vivo* validation to accelerate the translation of saffron-derived formulations into practical antimicrobial therapeutics.

Moving forward, more work is needed to understand the precise mechanisms that underlie these synergistic effects. It will also be important to assess the stability, safety, and performance of these optimized mixtures in more complex biological systems. Such efforts could pave the way for new, nature-inspired antimicrobial formulations with potential applications in healthcare and food preservation.

## Data Availability

The raw data supporting the conclusions of this article will be made available by the authors, without undue reservation.
